# An Interesting Adventure

**Published:** 1892-06

**Authors:** 


### AN INTERESTING ADVENTURE.

A correspondent of the *Medical and Surgical Reporter* tells about an exceedingly interesting adventure which happened to a Chicago physician during the congress at Berlin. The Illinois man passed over the Linden late at night reflecting upon the glory in store for his native city. Suddenly a suspicious looking individual hustled by, closely touching the doctor, the latter with the ingenuity of a Western man, felt for his watch, and missing it, unhesitatingly began the pursuit of the robber. The suspicious individual fled through the Brandenburg gate into the Thiergarten, the American doctor in close pursuit, loudly crying, "Put up that watch!" Near the Victory column the robber was caught by the doctor, and compelled to deliver the watch, after which he was released. The Chicago man returned to his hotel proud of himself and of his native city and country. But lo! On the table he beheld his watch, which he had forgotten on leaving the hotel. Next morning all the papers published the story of a robbery in the Thiergarten. A French doctor, they said, had been pursued by a burly robber, attacked and robbed of his valuable watch.—*Ex.*

